# Exploring the microbial communities in coastal cenote and their hidden biotechnological potential

**DOI:** 10.1099/mgen.0.001382

**Published:** 2025-04-03

**Authors:** Perla A. Contreras-de la Rosa, Susana De la Torre-Zavala, Aileen O´Connor-Sánchez, Alejandra Prieto-Davó, Elsa B. Góngora-Castillo

**Affiliations:** 1 Unidad de Biotecnología, Centro de Investigación Científica de Yucatán, Calle 43 No. 130. Col. Chuburná de Hidalgo, 97205, Mérida, Yucatán, México; 2 Facultad de Ciencias Biológicas, Instituto de Biotecnología, Universidad Autónoma de Nuevo León, 66425, San Nicolás de los Garza, Nuevo León, Mexico; 3 Unidad de Química-Sisal, Facultad de Química. Universidad Nacional Autónoma de México, 97356, Sisal, Yucatán, México; 4 CONAHCYT- Unidad de Biotecnología, Centro de Investigación Científica de Yucatán, Calle 43 No. 130. Col. Chuburná de Hidalgo 97205, Mérida, Yucatán, México; 5 CONAHCYT-Departamento de Recursos del Mar, Centro de Investigación y de Estudios Avanzados del Instituto Politécnico Nacional, Km 6. Antigua carretera a Progreso. Cordemex, 97310, Mérida, Yucatán, México

**Keywords:** biosynthetic gene clusters, coastal cenotes, metagenomics, secondary metabolites

## Abstract

Bacterial secondary metabolites are crucial bioactive compounds with significant therapeutic potential, playing key roles in ecological processes and the discovery of novel antimicrobial agents and natural products. Cenotes, as extreme environments, harbour untapped microbial diversity and hold an interesting potential as sources of novel secondary metabolites. While research has focused on the fauna and flora of cenotes, the study of their microbial communities and their biosynthetic capabilities remains limited. Advances in metagenomics and genome sequencing have greatly improved the capacity to explore these communities and their metabolites. In this study, we analysed the microbial diversity and biotechnological potential of micro-organisms inhabiting sediments from a coastal cenote. Metagenomic analyses revealed a rich diversity of bacterial and archaeal communities, containing several novel biosynthetic gene clusters (BGCs) linked to secondary metabolite production. Notably, polyketide synthase BGCs, including those encoding ladderanes and aryl-polyenes, were identified. Bioinformatics analyses of these pathways suggest the presence of compounds with potential industrial and pharmaceutical applications. These findings highlight the biotechnological value of cenotes as reservoirs of secondary metabolites. The study and conservation of these ecosystems are essential to facilitate the discovery of new bioactive compounds that could benefit various industries.

**Impact Statement**

Underground aquatic ecosystems, like cenotes, have been relatively understudied in terms of microbial biodiversity and their potential to produce bioactive compounds. This study marks an initial step towards exploring the genetic diversity and biotechnological potential of micro-organisms found in coastal cenote sediments. By employing metagenomic analyses, we identified diverse biosynthetic gene clusters involved in polyketide production, such as ladderanes and aryl-polyenes. Further, we conducted in-depth bioinformatics analyses to identify possible functions within the microbial community revealing that cenote micro-organisms may synthesize a variety of previously unknown bioactive compounds, such as antibiotics. The production of polyketides in underground environments remains poorly understood, limiting our knowledge of the biotechnological potential these ecosystems pose.

## Data Summary

The authors confirm all supporting data, code and protocols have been provided within the article or through supplementary data files. Seven supplementary figures and five supplementary tables are available with the online version of this article. Metagenome assemblies generated in this study have been deposited in the Sequence Read Archive (SRA) of the National Center for Biotechnology Information (NCBI) under the BioProject accession number PRJNA1167309.

## Introduction

Bacteria produce secondary metabolites, which are bioactive molecules that enable them to thrive in a given environment. Secondary metabolites serve a variety of functions, including communication, cooperation and competition. These molecules are utilized by bacterial communities to facilitate adaptation within their respective ecosystems [1,8].

Secondary metabolites are synthesized from organized groups of genes called biosynthetic gene clusters (BGCs), which vary in number according to the size of the bacterial genome [9,10]. In the fields of biotechnology and pharmacology, bioactive molecules are of significant interest due to their potential as the basis for the development of drugs or therapeutic products, given their antibacterial, antifungal and anticancer properties [11,17].

Currently, significant challenges in biotechnology, such as the rising prevalence of antibiotic-resistant bacteria and the growing demand for environmentally friendly products, underscore the necessity for the discovery of novel natural products that can more effectively address human needs [18,21]. The traditional methods for obtaining these metabolites include screening, mutagenesis and directed biosynthesis using precursors [22]. However, the advent of next-generation sequencing technologies has facilitated the acquisition of knowledge and the identification of secondary metabolites through metagenomics and bacterial genome sequencing [22,23].

Under-explored or extreme environments, such as water bodies, are sources of new information, both for the discovery of new bacterial strains and new BGCs [24]. For example, in the study by Zothanpuia *et al*. [25], actinobacterial strains encoding BGCs of antimicrobial bioactive compounds were isolated from freshwater sediment samples. Cuadrat *et al*. [26] identified BGCs by metagenomic analysis encoding secondary metabolites of pharmaceutical interest of the non-ribosomal peptide (NRP) and polyketide type from lake water [26].

**Fig. 1.**
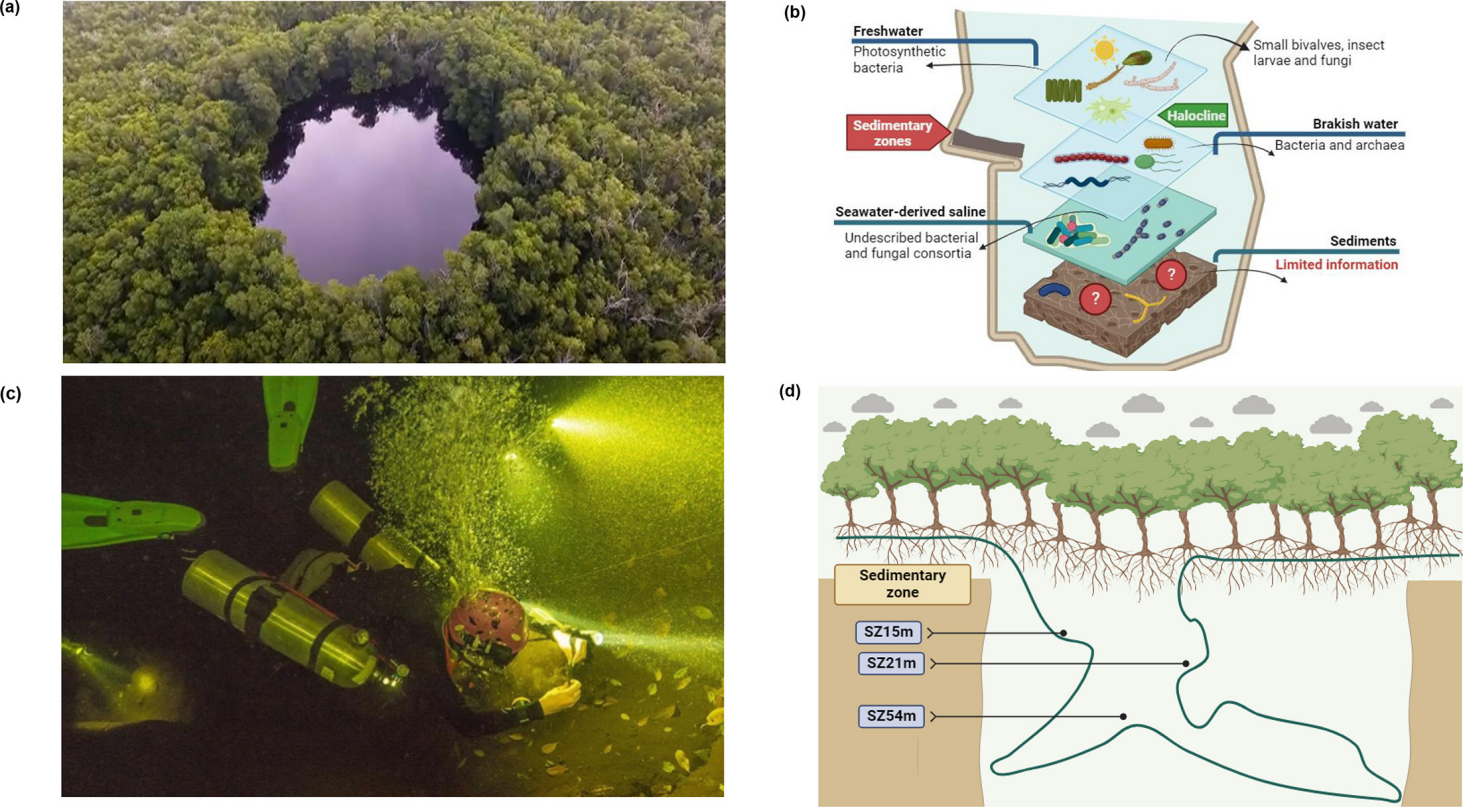
Sampling area and structure of the cenote water column. (a) Aerial photograph of cenote Pol-Ac. (b) Schematic representation of microbial communities in the cenote water column. (c) Sediment sampling in the cenote Pol-Ac. (d) Schematic representation of the underground cave structure and the sedimentary zones (SZs) of cenote Pol-Ac that were sampled.

In this context, Mexico’s Yucatán Peninsula emerges as a particularly promising environment, and only a few studies have analysed sediments in cenotes or natural water-filled sinkholes, for the identification of new compounds (Fig. 1a). The Yucatán Peninsula is an extensive limestone platform located in the southwestern region of Mexico. It is home to one of the world’s most extensive and important karst aquifers [27,28]. The region is composed of carbonate soil, which is soluble and compact. The karstification process creates conduits and caverns that facilitate the flow and storage of groundwater in caves known as sinkholes or ‘cenotes’ (derived from the Mayan term ‘ts'onot’) (Fig. 1a). The cenotes are dynamic spaces that interact with marine, interstitial and precipitation waters, creating diverse ecological niches that support endemic plant and animal species [28,30].

The available information on sinkholes is primarily focused on their morphology and hydraulic conditions, the fauna and flora, as well as archaeological remains and contamination [28,31,47]. There are only a few studies that focus on identifying the microbial communities inhabiting cenotes [48,51] and the secondary metabolites derived from these micro-organisms [52,55]. Recent studies have explored the biotechnological potential of microbial communities in cenotes. Wissner *et al.* [56] identified 49 Gram-positive bacterial isolates from coastal cenote sediment, belonging to various genera within the *Bacillota* and *Actinomycetota* phyla. These isolates demonstrated diverse enzymatic activities and antimicrobial properties against pathogens [56]. Similarly, Suárez-Moo and Prieto-Davó[55] employed genome mining techniques to analyse the biosynthetic potential of sediment microbial communities in a coastal cenote. They identified 203 BGCs and 55 secondary metabolites within 35 high-quality metagenome-assembled genomes (MAGs). Notably, 75 % of the BGCs showed no sequence homology with previously reported bacterial BGCs, suggesting potential novel compounds [55]. Both studies highlight the cenote sediments as promising sources for new natural products with applications in medicine and biotechnology, as well as compounds that may play significant ecological roles in these unique environments.

The cenote’s water column is composed of distinct aquifer layers (Fig. 1b). The upper water layer is composed of fresh water, with a varying thickness observed in different cenotes [49]. The following layer is saline water, which remains stratified due to the density difference between the freshwater and saline waters, creating a mixing zone known as the halocline [49]. The layer at the cenote bottom is composed of anoxic marine and transparent water [29]. Cenotes receive external organic matter (OM), which promotes the development of bacterial and fungal consortia [30]. The suspended OM is transported by slow flow into the subterranean galleries, where it is deposited in various sedimentary zones (SZs) and at the cenote bottom. The structural composition of SZ with OM may vary according to the characteristics of the cenote; therefore, the bacterial and archaeal communities present may differ depending on the environmental context [29,48, 49] (Fig. 1b).

The present study analysed three SZs of the coastal cenote ‘Pol-Ac’ located in the Yucatán Peninsula, Mexico. Using metagenomic approaches, we analysed the composition of bacterial and archaeal microbial communities and identified BGCs with biotechnological potential. Our findings led to the identification of polyketide synthase-type BGCs, including those associated with ladderanes and aryl-polyenes. Structural and genetic bioinformatics analyses suggest that these BGCs may be involved in the synthesis of novel active molecules, such as antibiotics. This study highlights the importance of exploring microbial communities in distinct SZs of the Pol-Ac cenote. This knowledge will facilitate current and future bioprospecting strategic studies as these understudied environments may serve as reservoirs of unknown secondary metabolites with industrial and pharmacological properties.

## Methods

### Study site and sampling

Sediment samples were collected in April 2021 from the coastal cenote Pol-Ac (21° 04′ 53.36" N, 90° 12′ 11.12" W), located within the ‘El Palmar’ nature reserve, a protected natural area in Yucatán, Mexico where human activities are restricted (Figs 1a and S1a, available in the online Supplementary Material). The Pol-Ac cenote is located ~800 m from the seashore (Fig. S1b) and is part of the ‘ring of cenotes’, a unique hydrological system in Mexico and globally. This system comprises a complex network of highly permeable caverns that conduct large volumes of groundwater flowing from southeast to northeast. Pol-Ac is an open cenote surrounded by coastal vegetation and mangroves, with a stratified water column reaching a depth of 63 m (Fig. 1d). The halocline, a zone of salinity gradient, is located at ~20 m depth.

Parameters such as temperature (°C), practical salinity unit (PSU), dissolved oxygen (mg l^−1^), pH and total suspended solids (TDS mg l^−1^) were measured *in situ* with a water EXO1 multiparameter probe from Xylem Analytics (Norway). The measurements were taken every 3 s along the water column.

Sediment samples were collected from three distinct SZs (Fig. 1c, d). Sample SZ15m was collected in the freshwater layer, from sediments located at 15 m below the surface. Sample SZ21m was collected from the halocline zone at a depth of 21 m. Sample SZ54m was collected from the cenote bottom at a depth of 54 m. The sediments were generally characterized by brown pigmentation and a smooth texture (Fig. S1). Sample SZ15m predominantly consisted of fine-grained soil, SZ21m exhibited a coarse-grained soil composition with rock fragments and shells and SZ54m was mainly composed of medium-grained soil (Fig. S1c-e). Each sample was collected in triplicate using hermetically sealable plastic bags with a capacity of 1 l and stored at 4 °C until the metagenomic DNA isolation.

### Quantification of total organic carbon present in sediment samples collected

To quantify the total organic carbon (TOC) in the sediment samples, 30 g of sediment was dried at room temperature for each sample. Samples were ground with an agate mortar and sieved with a 250 μm sieve. Approximately 1 g of sediment was treated with 1 N HCl, washed with deionized water and dried in an oven at 50 °C for 48 h.

The acidified and desiccated samples were weighed and encapsulated in silver capsules for subsequent analysis. The TOC was determined by combustion analysis using a COSTECH ECS 4010 elemental analyser. The percentage of carbon in the samples was calculated using a calibration curve of acetanilide (a known concentration standard) and reported in wet percentge (wt%) (Table 1).

**Table 1.** Physicochemical variables of the water column in SZs sampled from the Pol-Ac cenote, TOC in the sediments and general analysis of the metagenomic sequences obtained

Sample	Water column parameters				TOC in sediments	Metagenomic sequences	
**ID**	**Temp**.(**°C**)	**Salinity**(**PSU**)	**DO**(**mg** l^−1^)	**pH**	(**%**)	**No. of reads**(**M**)	**No. of contigs**(**K**)
**SZ15m**	26.1	38.3	0.04	6.99	11.70	27	98.8
**SZ21m**	26.4	38.7	0.07	6.97	7.88	24	38.8
**SZ54m**	26.7	39.4	0.09	6.96	16.46	22	64.4

* The values expressed represent the mean percentage by weight (wt. %) of the three sediment samples by sedimentary zoneSZ. The mean is 12.01%, with a standard deviation of 4.29.

DO, dissolved oxygen; ID, sample identifier; K, thousandsM, millions

### Metagenomic sequencing and data availability

Metagenomic DNA was isolated from 250 mg of sediment using the ZymoBIOMICS™ DNA Miniprep Kit (No. D4304), specific for metagenomic analyses. The DNA was isolated in triplicate for each sediment bag, and a composite sample was created for each depth.

Metagenomic sequencing was conducted by Novogene Co., Ltd. (CA, USA) using the Illumina NovaSeq 6000 platform in paired-end mode with a read length of 150 bp. The resulting data were deposited in the NCBI SRA under Biosample accession numbers SAMN43994686, SAMN43994687 and SAMN43994688.

The bioinformatics analyses were conducted on a high-performance computing cluster with a Red Hat Enterprise Linux (RHEL) (v7.1) operating system.

### Quality control, assembly and taxonomic assignment

Read quality was assessed using FastQC (v0.11.5) with default parameters [57]. High-quality reads were annotated both functionally and taxonomically using the MG-RAST server (v4.0.3) [58]. Prokaryotic reads were selected for the analysis and visualization of microbiome census data using the Phyloseq package in R (v4.2.1) [59]. Total high-quality filtered reads were selected for downstream analyses and assembled using Megahit (v1.1.4), with a minimum contig size threshold of 500 bp [60]. The data have been deposited in the NCBI SRA under Biosample accession numbers: SAMN43994689, SAMN43994690 and SAMN43994691.

MetaBAT2 (V2.15) was employed to obtain MAGs, using the default parameters and including only contigs larger than 1,500 bp [61].

The completeness and contamination of the MAGs were assessed with the CheckM program (v1.1.3) [62], which uses bacterial marker genes for validation. MAG quality was classified according to the standards established by the Genomic Standards Consortium. High-quality MAGs have a completeness of 90% or higher and contamination of 5% or lower. Medium-quality MAGs exhibit a completeness of at least 50%, with contamination under 10%, while low-quality MAGs have less than 50% completeness and contamination of 10% or less [63]. Taxonomic annotation of MAGs was carried out using GTDB-Tk with default parameters (v2.4.0) [64], using the Galaxy platform (https://usegalaxy.org/) [65].

### Analysis of microbial community structure

The alpha diversity and the Pielou evenness index (J’) [66] were calculated using the Phyloseq package in R (v4.2.1) [59], considering reads previously identified as bacterial and archaeal genera by MG-RAST. The dissimilarity between microbial community compositions across the three different SZs was evaluated using the Vegan package (v2.6–4) in R [67] with a Bray–Curtis distance analysis [68].

### Detection of BGCs and identification of metabolic pathways

The antiSMASH (v6.0) program was used in strict mode to identify complete BGCs in the assembled contigs [69]. Contigs containing BGCs were taxonomically annotated using the non-redundant NCBI database [70]. The BlastKoala tool (http://www.kegg.jp/blastkoala/) was used to identify metabolic pathways [71,72].

### Detection, analysis and functional annotation of novel BGCs

The novel BGCs were identified using the BiG-SLiCE tool [73] and the BiG-FAM database (v1.0.0) [74] with a Euclidean distance cutoff of 900 [74]. The core genes that include ketosynthase (KS) domain sequences were selected with antiSMASH, and a phylogenetic inference was conducted. Known BGCs were obtained from the results of the ‘MiBiG comparison’ section of the antiSMASH report [75].

Multiple sequence alignment was conducted using the online tool MAFFT (v7) [76] (https://mafft.cbrc.jp/alignment/server/index.html). Phylogenetic trees were built using the online server PhyML [77] (http://www.atgc.montpellier.fr/phyml/), with the next parameters: (i) the Bio-Neighbor Joining (BioNJ) algorithm, (ii) the subtree pruning and regrafting tree rearrangement method and (iii) the approximate likelihood ratio test (aLRT SH-like) to support the branches. Branch support values are expressed as percentages. External groups included terpene/carotenoid-type BGCs such as *Algoriphagus* sp. (MiBIG accession no. BGC0000650), *Brevundimonas* sp. (MiBIG accession no. BGC0000634) and type III polyketide synthase (PKS-III) from *Mycobacterium marinum* (MiBIG accession no. BGC0001665). The colours and names of the phylogenetic trees were edited using the mega programme (v11) [78].

## Results and discussion

### *In situ* evaluation of the cenote water column variables and metagenomic sequence data from the cenote sediments

The temperature, salinity and dissolved oxygen were measured along the cenote water column, between 0 and 54 m depth (Fig. 1d). The temperature ranged from 27 °C at the surface to 26 °C at 54 m depth. Salinity values spanned from 0 to 39 practical salinity units, with the halocline located at 21 m depth. Finally, dissolved oxygen values exhibited a decrease from 2.66 to 0.01 mg l^−1^ with depth (Fig. S2).

Metagenomic DNA was obtained from three SZs (SZ15m, SZ21m and SZ54m) and sequenced using the Illumina platform, resulting in 27, 24 and 22 million 150 bp reads, respectively (Table 1).

### Microbial community composition identified in sediment metagenomes

The microbial community structure was analysed using MG-RAST [58]. High-quality reads and contigs derived from the assembly of each metagenome were annotated by domain. The results revealed that bacterial sequences comprised 93, 85 and 90% of the SZ15m, SZ21m and SZ54 metagenomes, respectively, while Archaea sequences ranged from 6 to 13%. Sequences identified as eukaryotes, viruses and unclassified sequences each accounted for less than 1% (Fig. S3).

**Fig. 2.**
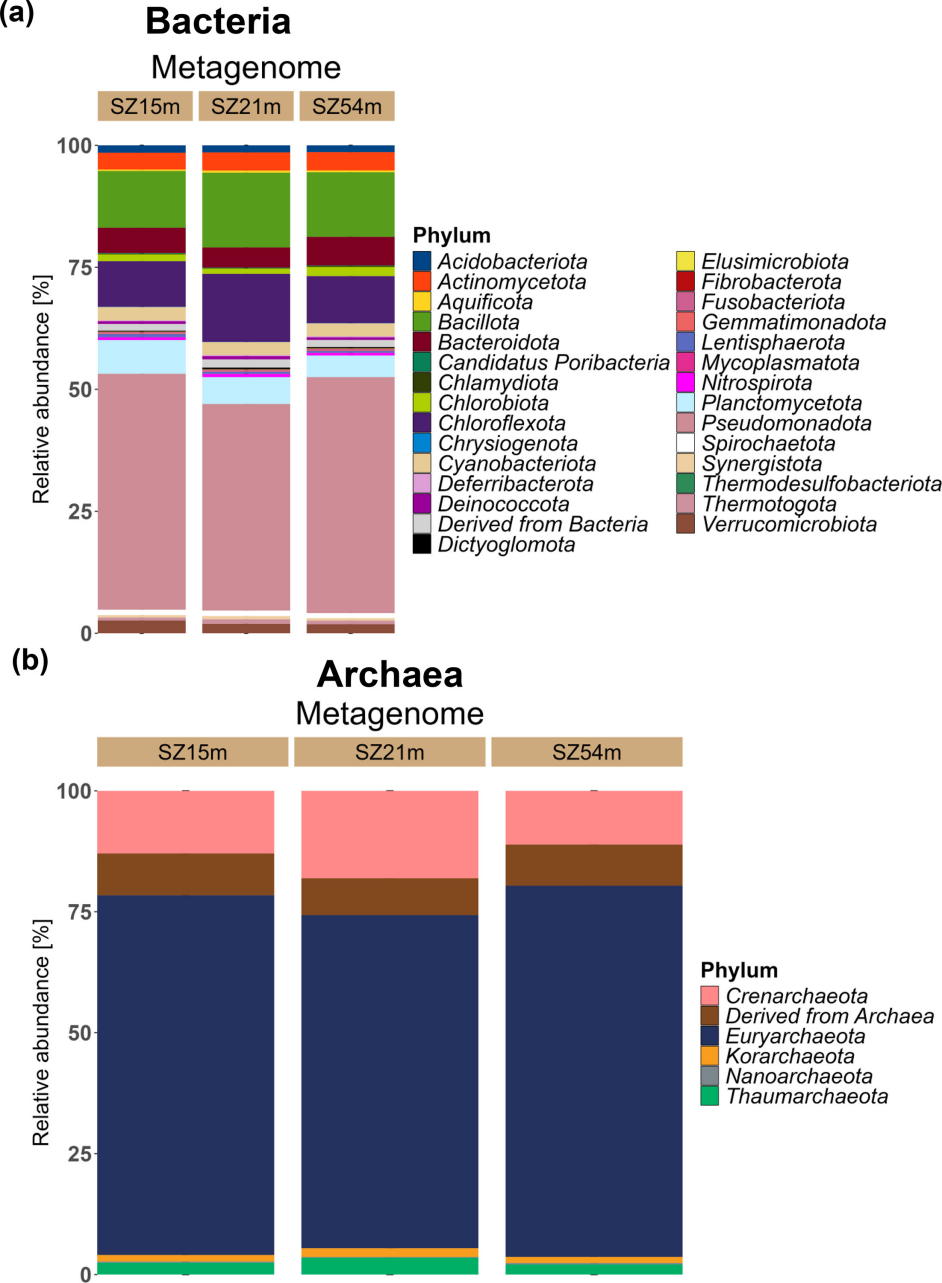
Relative abundances of microbial communities at the phylum level across SZs from the Pol-Ac cenote. Reads identified as derived from the domains Bacteria and Archaea were selected based on MG-RAST annotations to calculate the relative abundances of various phyla. (**a**) Distribution of 35 Bacterial phyla. (**b**) Distribution of six Archaeal phyla.

Bacteria and Archaea sequences were selected to assess the community composition in each metagenome. A combined analysis of the three metagenomes identified a total of 35 phyla, including 29 bacterial and six archaeal phyla. Among these *Pseudomonadota* was the most dominant phylum (45%) followed by *Bacillota* (25%), *Chloroflexota* (20%) and *Planctomycetota* (10%) (Fig. 2a). These findings are consistent with previous studies, which report that these phyla are highly abundant in coastal and inland cenotes, as well as in mangrove environments [48,79]. At this taxonomic level, the bacterial composition of Pol-Ac cenote sediments appears to align closely with that of other known cenotes, showing no major differences.

Likewise, the predominant Archaea phyla in each of the three metagenomes were *Euryarchaeota* (75%), followed by *Crenarchaeota* (20%) and a group classified as ‘Derived from Archaea’. The phyla *Korarchaeota*, *Thaumarchaeota* and *Nanoarchaeota* exhibited the lowest abundance, representing ∼5% of the relative abundance each (Fig. 2b). The occurrence of archaea in the sediments of cenotes has not yet been fully elucidated. Nevertheless, previous research showed that the predominant phylum in some coastal and inland cenotes sediments is *Euryarchaeota* [48,49]. Additionally, Gómez-Acata *et al*. [79] identified the phyla *Thaumarchaeota*, *Bathyarchaeota* and *Nanoarcheota* in mangrove-interacting sediments. Thus, our results were consistent with previous studies, identifying the predominant bacterial and archaeal phyla, suggesting that cenote sediments have a structured microbial community regardless of location and cenote-specific characteristics.

Notably, sulphate-reducing bacteria and Archaea (SRB) were identified in all three communities, including members from the Deltaproteobacteria, *Bacillota*, *Euryarchaeota* and *Crenarchaeota* groups (Fig. 2b). These groups were particularly prevalent in metagenome SZ21m, which corresponds to the halocline zone. This zone is characterized by the presence of hydrogen sulphide (H_2_S), an odourless gas that is the end product of the majority of sulphur oxidation/reduction pathways. The reduction of sulphur is exclusively mediated by sulphate-reducing bacteria (SRB) bacteria and archaea, which oxidize OM by using sulphate and produce H_2_S as a by-product [37,49, 80].

Microbial communities undergo restructuring in response to the environmental conditions of their habitat [81,83]. This dynamic has been observed in comparative studies of coastal and inland cenotes, wherein it has been demonstrated that variations in salinity and temperature along the water column affect the composition of these communities [48]. In the case of the Pol-Ac cenote, the salinity and temperature conditions remained practically constant throughout the water column (Fig. S2), which may explain the uniformity of the bacterial and archaeal communities in the three SZs (Fig. 2).

### Biodiversity metrics of microbial communities of sampled sediments in the Pol-Ac cenote

Karstic aquifers are characterized by their porosity type and reduced water flow velocities, resulting in prolonged water residence times compared with other types of aquifers. This dynamic implies a lack of constant water renewal. As a result, microbial communities in water-saturated sediments are generally stable in terms of biomass and diversity [84]. This stability was evident in the three metagenomes, which exhibited high genus diversity with a relatively uniform distribution, as indicated by the Shannon index (5.29–5.35) [66] (Fig. S4). Chao1 values indicated that the estimated richness of genera was only slightly higher than observed, confirming that the sampling effort was sufficient to identify most of the genera present in each metagenome community [85] (Table 2).

**Table 2.** Ecological indices of prokaryotic communities of sampled sediments in the Pol-Ac cenote

Metagenome	No. of genera	Chao1	Shannon (H’)	Evenness (J)
SZ15m	900	1,100	5.30	0.78
SZ21m	871	932	5.35	
SZ54m	868	938	5.29	

1, estimated richness; Evenness, Pielou’s evenness index(H’), Shannon diversity index

However, the Bray–Curtis distance metric, which measures dissimilarity between microbial communities, revealed that the archaeal and bacterial genera in SZ21m exhibited a higher degree of dissimilarity compared with SZ15m and SZ54m (Fig. S5). This difference can be attributed to the unique characteristics of SZ21m as a halocline zone, characterized by the lowest dissolved organic carbon levels (7.8%) compared with SZ15m (11.7%) and SZ54m (16.46%) (Table 1). The limited availability of dissolved organic carbon may favour the proliferation of archaea. The increased abundance of archaea in this zone could drive a higher rate of sulphur oxidation reactions, further differentiating its microbial community. Previous studies have suggested that organic carbon levels can impact microbial community structure by influencing both the density and diversity of these communities [86,87].

Additionally, the sediments in SZ21m contain shells and small stones, features absent in the other SZs, which might also contribute to its microbial composition (Fig. S1) [81]. In contrast, Pol-Ac is an open cenote surrounded by mangrove vegetation (Fig. 1a), where there is a constant input and degradation of OM [29]. This deposited material accumulates in various SZs, both along the cenote walls and the bottom of the cenote, resulting in higher TOC accumulation in the floor sediments (SZ54m) compared with upper zones (SZ15m and SZ21m) (Table 1).

Overall, these results suggest that the stability of the water column, reflected in constant physicochemical factors such as pH and temperature, contributes to homogeneous microbial communities across the different zones of the Pol-Ac cenote. Furthermore, the availability of dissolved organic carbon is a key factor in shaping the microbial community composition in the SZs of the Pol-Ac cenote.

### Metagenome-assembled genomes

A total of four MAGs with different degrees of completeness were obtained (Table S1). Three of these MAGs (1SZ15m, 2SZ15m and 3SZ15m) were identified in the SZ15m metagenome, while one (4SZ54m) was found in the SZ54m metagenome (Table 3). The number of contigs per MAG varied from 73 to 1,175 for the MAGs 1SZ15m, 2SZ15m and 3SZ15m, and 303 contigs for MAG 4SZ54m. The sizes of the MAGs ranged from 240 kb to 6 Mb, and the predicted gene count varied from 337 to 6,645. However, completeness analyses classified only MAGs 3SZ15m and 4SZ54m as medium quality, with completeness of 30 and 40%, respectively, and no detectable contamination.

**Table 3.** Genome information obtained from the MAGs

MAG ID	Contig	Genome size(Kb)	Completeness(%)	Contamination(%)	No. of predicted genes	BGC	Taxonomy classification	Quality
**1SZ15m**	100	310	13	0	337	None	Unclassified Bacteria	Low
**2SZ15m**	1,175	6,000	90	64	6,645	None	*Anaerolineae*	Low
**3SZ15m**	73	240	30	0	342	None	*Dehalococcoidales*	Medium
**4SZ54m**	303	1,000	40	0	1,069	Yes	*Desulfatiglandales*	Medium

Taxonomic annotation was performed using GTDB-Tk [64], with the relative evolutionary divergence metric. The results indicated that all four MAGs correspond to the Bacteria domain (Table 3). However, these findings should be interpreted with caution due to the low completeness of the recovered MAGs.

For this study, we focused on medium-quality MAGs to examine the prevalence of BGCs, and those classified as low quality were excluded from the BGC analysis. Notably, no MAGs were recovered from the SZ21m metagenome, which consisted of contigs ranging from 1 to 5 kb. In contrast, the other metagenomes had contigs sizes ranging from 1 to 50 kb. Furthermore, we are able to identify a 2.59 kb aryl-polyene BGC in MAG 4SZ54m, accounting for 0.25% of the total MAG size (Table 3).

The number of BGCs in bacterial genomes typically correlates with genome size and taxonomic classification. For instance, the phylum *Cyanobacteriota* averages 3.4 BGCs per genome, while Acidobacteria, *Pseudomonadota*, *Nitrospirota*, *Bacillota* and *Planctomycetota* typically exhibit ~1.4 BGCs per genome [88]. Consistent with this trend, the aryl-polyene BGC in MAG 4SZ54m was classified within the phylum *Pseudomonadota*, aligning with the expected BGC for this group. However, genus-level identification was not possible, likely due to the incompleteness of the genome or the absence of a well-characterized reference genus.

The analysis of MAGs has significantly enhanced our understanding of bacteria and archaea, particularly those that are challenging to cultivate, and offers a powerful tool for BGC identification [89]. Recovering BGCs associated with secondary metabolite production provides valuable insights into the metabolic and ecological functions of microbial communities in their natural habitats, while also revealing potential applications for these metabolites [26,90]. In this study, the identified BGC was analysed using antiSMASH [69], which revealed an 8% similarity to an aryl-polyene-type BGC related to flexirubin biosynthesis, a class of orange pigments. However, these findings should be interpreted with caution due to the very low similarity, which limits the certainty of functional predictions and suggests the possibility of novel or divergent pathways.

### Metabolic pathway analysis and BGCs detected in metagenomes

Using the MG-RAST web tool, archaeal and bacterial contigs were annotated with the Kyoto Encyclopedia of Genes and Genomes (KEGG) orthology database (Fig. S6). The analysis revealed a similar distribution of metabolic pathways across the three metagenomes, with 14 distinct metabolic subsystems identified. Among these, carbohydrates represent ~13% of the sequences, followed by clustering-based subsystems (12%), amino acids and derivatives (10%), protein metabolism (8%), miscellaneous function (7%), cofactors, vitamins, prosthetic groups and pigments (6%), metabolism (6%), fatty acids, lipids and isoprenoids (6%), respiration (5%), DNA metabolism (4%), fatty acids, lipids and isoprenoids (3–5 %), cell wall and capsule (3%), membrane transport (2.5%), nucleosides and nucleotides (2.5%) and stress response (2%) (Fig. S6).

The metabolic analysis provides a comprehensive overview of the functional potential of the microbial communities within the metagenomes. Building on this, we explored their biosynthetic potential by identifying BGCs in the Pol-Ac cenote using antiSMASH [69]. As a widely used tool, antiSMASH predicts cluster types and potential metabolite classes using sequence similarity-based methods to detect core biosynthetic enzymes and predict BGCs for a wide range of secondary metabolites, including polyketides, non-ribosomal peptides and terpenes [91]. However, its predictions rely on pathway similarity, meaning structural diversity within BGCs of the same type can result in variations in biological activity. Therefore, further structural and functional validation is necessary to confirm the inferred activities and ensure accurate interpretation of the identified BGCs.

**Fig. 3.**
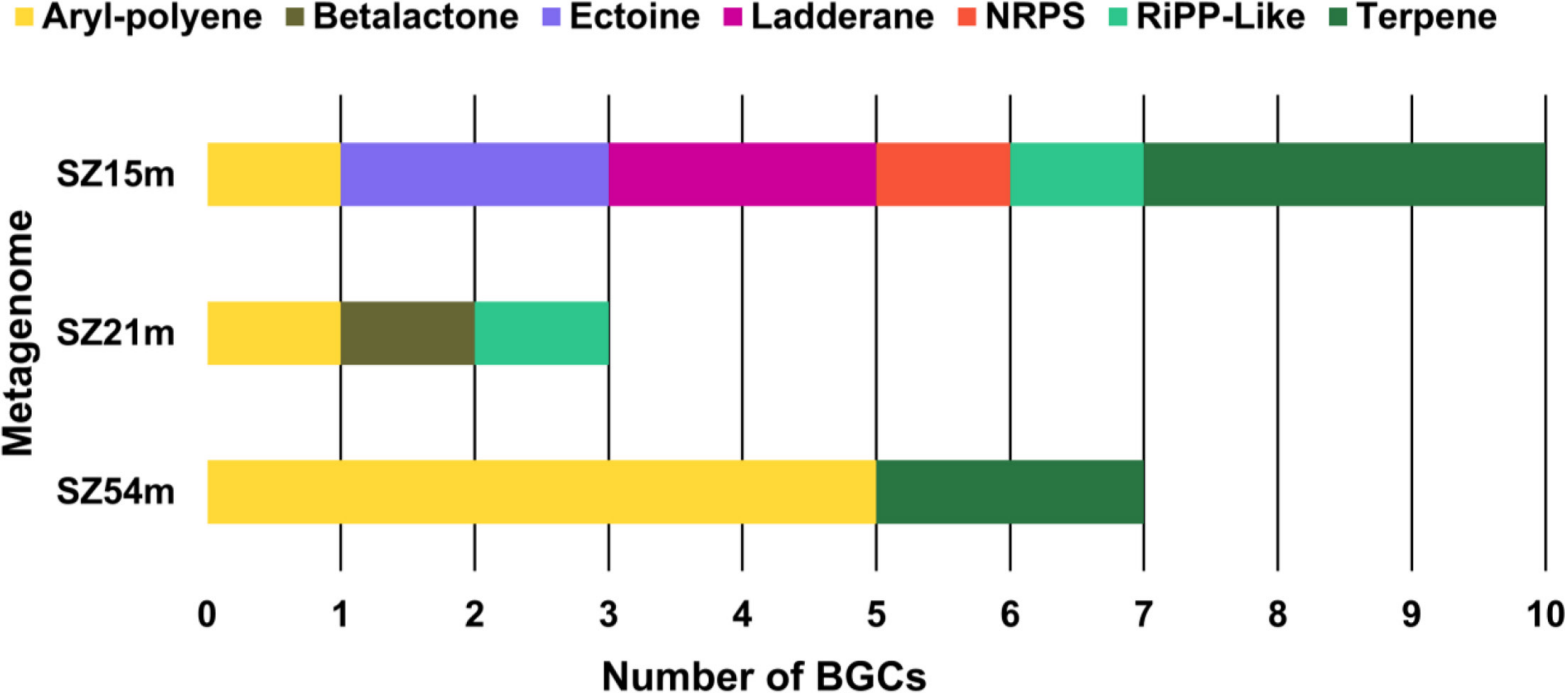
Number and types of BGCs identified in each metagenome. Individual bars correspond to individual metagenomes. Different colours in bars correspond to the seven BGC types identified and the abundance of each BGC type in each metagenome.

A total of 20 BGCs were identified within the contigs from the three assembled metagenomes (Table S2). Of these, ten were identified in metagenome SZ15m, three in SZ21m and seven in SZ54m. The identified BGCs were classified into seven different category types: (i) aryl-polyene, (ii) betalactone, (iii) ectoine, (iv) ladderane, (v) non-ribosomal peptide-NRPS, (vi) ribosomal synthesized and post-translationally modified peptides (Ripp-Like) and (vii) terpene type (Fig. 3).

1. Aryl-polyene type: A total of seven BGCs of the aryl-polyene type were identified. Two of these were in the SZ15m and SZ21m metagenomes, and the other five were in the SZ54m metagenome (Fig. 3). The aryl-polyenes are bacterial pigments that have been categorized into four distinct classes. (i) xantomonadina, (ii) arcuflavin, (iii) flexirubin and (iv) APE similar to flexirubin, which are synthesized via a type II polyketide synthase (PKS-II) pathway, analogous to the fatty acid biosynthetic pathway [92]. *In vitro* studies have demonstrated that these pigments contribute to protection against photodamage caused by visible light and reactive oxygen species and play a role in biofilm formation [93,97]. Interestingly, one of these BGCs was within a contig classified under the phylum *Uroviricota* (uncultured *Caudovirales* phage; *E*-value≤1×10^−3^, identity 79.35%), indicating its potential association with a prophage. Previous studies have established a link between prophages and BGCs encoding bacteriocins, which are involved in bacterial defence mechanisms. For instance, Dragoš *et al.* [98] identified prophages harbouring BGCs that encode bacteriocins, proteins that destroy or inhibit the growth of other bacteria. Additionally, Achudhan *et al.* [99] identified CRISPR elements in bacterial and archaeal genomes via metagenomic analysis, suggesting interactions with *Caudovirales* phages, which typically infect commensal or pathogenic bacterial hosts. Considering the ecological functions attributed to aryl polyenes, it is likely that the identified BGC provides competitive advantages related to cellular protection, complementing previous findings on prophage-associated BGCs encoding bacteriocins [98].
2. Betalactone type: Two BGCs of this type were found in the SZ21m metagenome (Fig. 3). The products of betalactone-type BGCs are key in the formation of a wide range of chemical compounds, including terpenes and hybrid PKS/NRPS compounds. To date, 30 unique natural betalactone products have been identified. Notably, only eight BGCs of this type have been reported, and these have only been found in two phyla: *Actinomycetota* and *Pseudomonadota* [100]. In our findings, the contig containing the betalactone BGC could not be taxonomically classified, leaving the organism of origin unidentified. This underscores the importance of studying these environments to gain a deeper understanding of their microbial diversity.
3. Ectoine type: Ectoine is widely utilized by bacteria to protect against osmotic stress, extreme temperatures and damage caused by ionizing radiation to proteins and DNA [101,104]. Two ectoine BGCs, which could not be taxonomically classified, were detected in the metagenome of the surface zone (SZ15m) (Fig. 3). In contrast, Suárez-Moo and Prieto-Davó [55] identified ectoine BGCs in most cultivation-dependent metagenomes and MAGs derived from the SZ54m sample of the Pol-Ac cenote, specifically the genera *Halomonas* (*Pseudomonadota*) and *Virgibacillus* (*Bacillota*). These findings, along with our results, suggest that ectoine-type BGCs may play an adaptive role in high salinity conditions, as SZ15m (38.3 PSU) and SZ54m (39.4 PSU) exhibit similar salinity levels (Fig. S2).
4. Ladderane type: A novel putative BGC within a contig assigned to *Edwardsiella ictaluri* (*E*-value≤1×10^−5^, identity 91.49%), a bacterium from the phylum *Pseudomonadota*, was identified in the SZ15m metagenome (Fig. 3). The *Pseudomonadota* phylum also exhibited the highest abundance in the microbial communities (>50%) of the Pol-Ac cenote (Fig. 2a). Ladderanes are lipid components typically associated with the membrane of anaerobic ammonium oxidizing bacteria (*anammox*) [105]. However, the biochemical pathways responsible for their biosynthesis remain unknown [106,108]. Interestingly, ladderane BGCs have also been reported in non-anammox bacteria [109,111], and it is hypothesized that these clusters may play a role in fatty acid biosynthesis. To date, 34 genes have been identified as potentially involved in this biosynthetic process [112]. A method for their assembly has been proposed for expression in hosts such as *Escherichia coli*, with the goal of elucidating their biosynthetic pathway [108]. The discovery of ladderane BGCs in non-anammox bacteria raises important questions regarding their role in environmental adaptation. Research on ladderane BGCs is still in its early stages, and further investigation into metagenomes will be crucial for understanding the prevalence and function of these BGCs in non-anammox bacterial genomes.
5. NRP type: A 2.2 kb NRP BGC was identified in the SZ15m metagenome, and the contig containing this BGC was taxonomically classified as *Burkholderia glumae* (*E*-value≤5×10^−47^, identity 73%) (Fig. 3, Table S2). Although the specific roles of these secondary metabolites in microbial communities are not yet fully understood, it is hypothesized that in non-pathogenic strains, such as *Actinomycetota*, these metabolites may be involved in bacterial communication or signalling [113]. Additionally, these BGCs can facilitate mutualistic interactions between bacteria and organisms like algae and insects [114]. For instance, *Candidatus Endobryopsis kahalalidefaciens* establishes a symbiosis with *Bryopsis sp*. algae, by synthesizing kahalalides (toxins that protect algae) with its NRPS-type BGCs [115]. The genus *Burkholderia* (a member of the phylum *Pseudomonadota*) is known for its wide range of secondary metabolite-producing bacteria, particularly those with NRPS and PKS biosynthetic pathways and antimicrobial activity. The identification of new BGCs in *Burkholderia* species has advanced through genome analysis and using recombination systems [116,119]. Our study suggests that microbial communities from cenotes could be a potential source of novel NRPS BGCs.
6. Ribosomal synthesized and post-translationally modified peptide (Ripp-Like) type: In our results, two Ripp-like BGC were identified in the SZ15m and SZ21m metagenomes. Both BGCs included core and associated genes, with the SZ15m BGC measuring 1,015 nt and the SZ21m BGC measuring 1,820 nt. The contig containing the SZ15m BGC was taxonomically assigned to *Verrucomicrobiota* (*E*-value ≤2×10^−42^, identity 80%) (Table S3). This phylum is typically found in soil and sediment and is known for producing diverse BGCs involved in the synthesis of terpenes, aryl-polyenes, PKS, NRPS, as well as lichenans and betalactones [110,120, 121]. RiPP BGCs have genes encoding small ribosomally synthesized precursor peptides and enzymes that can modify these precursors, resulting in a high degree of structural diversity [122,123]. RiPPs have been shown to play diverse roles, including quorum sensing, interspecies competition, morphological development, host–microbe interaction and biofilm formation [124]. Interestingly, despite the relatively low abundance of *Verrucomicrobiota* (~5%) in our sample (Fig. 2a), a RiPP-type BGC of this bacterial group was identified, suggesting that BGC prevalence does not always correlate with phylum abundance. This emphasizes the importance of metagenomic analysis for uncovering potential new metabolites in less abundant or understudied bacterial groups.
7. Terpene type: Our analysis identified five terpene BGCs: three in the SZ15m metagenome and two in the SZ54m metagenome (Fig. 3). The contigs containing BGCs from SZ15m metagenome were classified as unknown uncultivated organisms (*E*-value ≤1×10⁻⁶⁵ identity 80%), *Streptomyces clavuligerus* (phylum *Actinomycetota*) (*E*-value ≤1×10⁻⁸, identity 85.33%) and *Anaerolineae bacterium* (phylum *Chloroflexota*) (*E*-value ≤1×10⁻²⁵, identity 72.14%). The actinobacteria *Streptomyces clavuligerus* produces valuable compounds like clavulanic acid and has been extensively studied as a heterologous system for the expression of bioactive compounds, including terpenes [125]. The two contigs with the BGCs from the SZ54m metagenome could not be taxonomically identified. Terpenes constitute the largest class of natural products and have various biotechnological applications [126,127]. However, their synthesis poses significant challenges due to their molecular complexity and the specific cultivation conditions required for production [128]. While most known terpenes come from plants and fungi, finding them in bacteria opens up new possibilities [128,130]. For instance, Chen *et al.* [131] found that terpenoid BGCs were abundant in deep microbial mats, even in low-light conditions, suggesting that photosynthetic bacteria, such as cyanobacteria, may be using these BGCs to adapt to light-deprived conditions. In contrast, in our study, the terpenoid BGCs were found in non-photosynthetic bacteria, suggesting that they may be playing different roles in this unique environment. The presence of the genus *Streptomyces* in the Pol-Ac cenote sediments is promising for the future discovery of new molecules.

Our findings highlight the critical role of metagenomics in identifying BGCs and their associated secondary metabolites. By offering a comprehensive view of the biosynthetic potential within microbial communities, metagenomics enables the development of targeted analytical approaches and accelerates the discovery of compounds with biotechnological applications.

### Identification and functional annotation of novel BGCs

The analysis using the BiG-SLiCE tool [73] on 20 BGCs identified five as novel BGCs (Euclidean distance>900) (Table S3), including three aryl-polyene (Abgc1_15 m, Abgc2_21 m and Abgc3_54 m) and two ladderane (Lbgc1_15 m and Lbgc2_15 m) (Table 4).

**Table 4.** Novel BGCs. The size of the arrows is proportional to the gene size, and the transcriptional direction is indicated by the orientation of each arrow. The colours follow the antiSMASH v6.0 colour scheme

Predicted BGC cluster	ID	Size (Kb)	Similarity score of the most similar BGC	Micro-organism of the most similar BGC(Phylum)	Euclidean distance	ID taxonomic NCBI contig(Phylum)	Identity annotated contig
**Aryl-polyene**							
	Abgc1_15 m	3.84	0.30	*Aliivibrio fischeri* ES114(*Pseudomonadota*)	1119	*Caudovirales phage*(Uroviricota)	79%
	Abgc2_21 m	1.23	0.27	*Moorea producens JHB*(*Cyanobacteriota*)	1042	na	na
	Abgc3_54 m	1.52	0.27	*Moorea producens JHB*(*Cyanobacteriota*)	1042	na	na
**Ladderane**							
	Lbgc1_15 m	1.35	0.48	*Streptomyces nigrescens*(*Actinomycetota*)	972	na	na
	Lbgc2_15 m	1.20	0.45	*Photorhabdus laumondii* subsp. *laumondii* TTO1(*Pseudomonadota*)	962	*Edwardsiella ictaluri*(*Pseudomonadota*)	91%

na, Not applicable.

Aryl-polyene BGCs, found in all three metagenomes, ranged in size from 1.23to 3.84 Kb and had two to eight genes. These BGCs showed similarity with BGCs found in *Aliivibrio fischeri* ES114 and *Moorea producens* JHB (similarity>0.25). Notably, the *Moorea* genus, a cyanobacterium, is known for its remarkable capacity to produce bioactive secondary metabolites [132]. In contrast, ladderane BGCs were found in the SZ15m metagenome, ranging in size from 1.21 to 1.35 Kb, and comprising two to three genes. Similar BGCs (similarity>0.4) were found in *Streptomyces nigrescens* and *Photorhabdus laumondii* subsp. *laumondii TTO1* genomes. Interestingly, *S. nigrescens* has been studied in co-culture with other actinobacteria and has been documented to produce compounds with antifungal and antimicrobial properties [133]. In addition, the genus *Photorhabdus* has been found symbiotically associated with nematodes and produces metabolites with insecticidal activity [134,135]. These findings are particularly significant, as tropical marine environments, such as the one studied, offer promising opportunities to isolate and study novel organisms, as well as organisms with similar metabolic profiles, which could accelerate the biodiscovery process.

Taxonomic annotations linked Abgc1_15 m to *Caudovirales phage* (*E*-value<1×10^−3^, identity 79.35%) and Lbgc2_15 m to *E. ictaluri,* a bacterium from the phylum *Pseudomonadota* (*E*-value <1×10^−5^, identity 91.49%) (Table 4). *E. ictaluri* is an aquatic pathogen known to infect the catfish *Ictalurus punctatus* [136], and to date, no reports have been published on its metabolic capabilities.

**Fig. 4.**
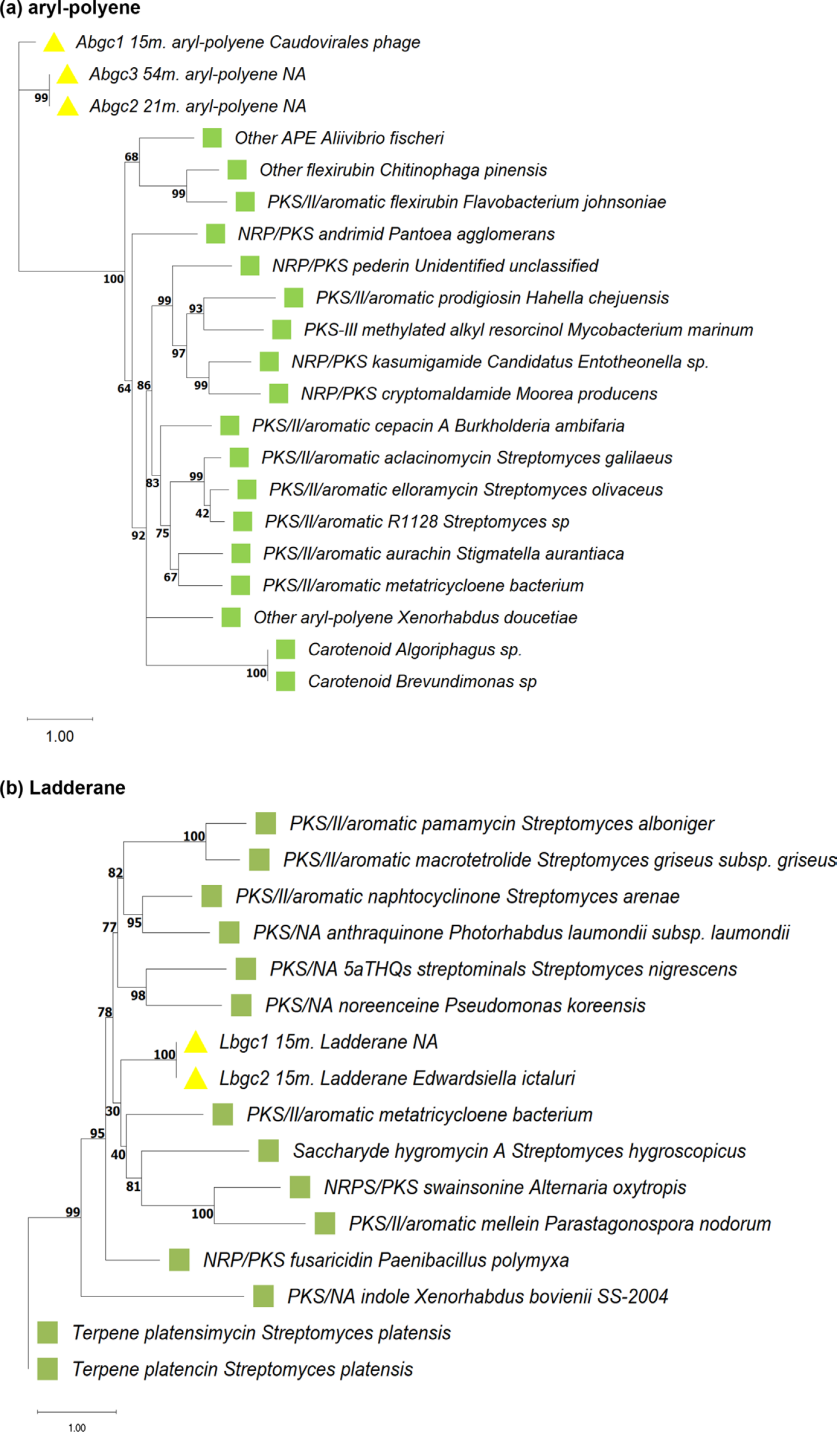
Phylogenetic inference of novel BGCs. (**a**) Phylogenetic tree of aryl-polyene BGCs; (**b**) phylogenetic tree of ladderane BGCs. Each tree includes both novel and known BGC core genes. The known BGC core genes include the KS domain sequences. Known core genes are highlighted with green squares, and novel core genes are indicated by yellow triangles. Branch labels specify BGC ID, metabolite type and the organism. Each branch is annotated to indicate whether it corresponds to a PKS-I or PKS-II type, with a further distinction between aromatic or linear. Scale bars represent evolutionary distance, and branch support values, calculated using the approximate likelihood ratio test (aLRT SH-like), are shown as percentages (0–100%).

To investigate the relationship between novel aryl-polyene BGCs (Abgc1_15 m, Abgc2_21 m and Abgc3_54 m) with known BGCs, a phylogenetic tree was constructed using core genes from six BGC types: NRP-PKS, PKS, NRPS, aryl polyene, flexirubin and terpene (Fig. 4a, Table S4). Phylogenetic analyses revealed that aryl-polyene BGCs formed a distinct clade, suggesting the synthesis of novel metabolites. The closest cluster included BGCs from *Streptomyces* species known for producing bioactive compounds like aclacinomycin and elloramycin, which exhibit anticancer and antimicrobial properties, respectively [137,139].

Similarly, a phylogenetic tree was constructed for novel ladderane (Lbgc1_15 m and Lbgc2_15 m) using core genes from NRP-PKS, PKS and terpene biosynthesis (Fig. 4b, Table S4). Ladderane BGCs clustered with those associated with metabolites, such as metatricycloene, hygromycin A, swainsonine and mellein, which have broad biological applications. For example, metatricycloene, a metabolite biosynthesized by PKS-type BGCs, is associated with carotenoids and antibiotic activity [140], swainsonine is a natural alkaloid with anticancer properties [141,145] and mellein is used as a larvicide, antifungal and antibacterial agent [146].

**Fig. 5.**
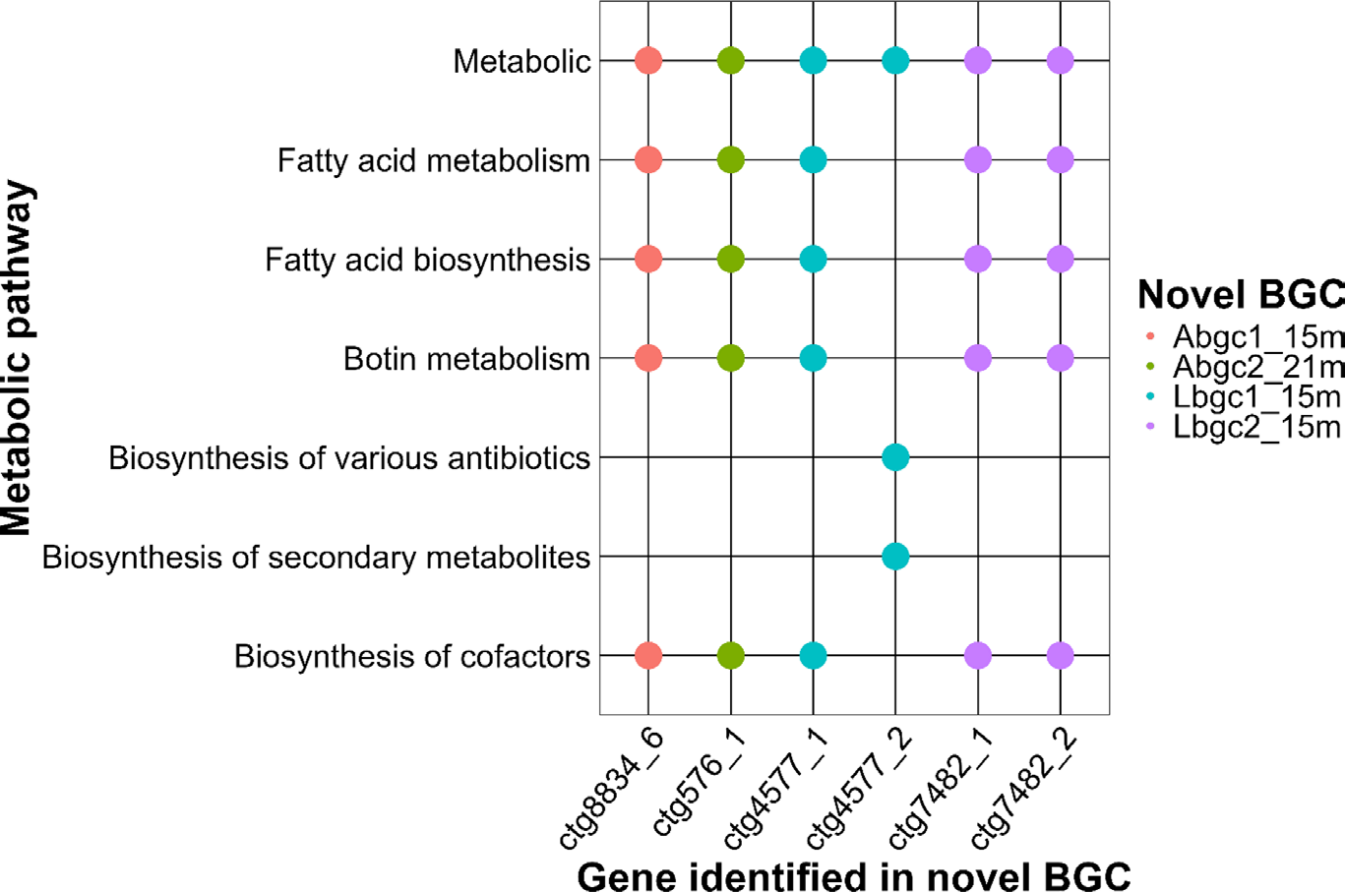
Biosynthetic cluster genes and associated metabolic pathways. Genes belonging to the new biosynthetic clusters (*x*-axis) were annotated using the KEGG database to identify the metabolic pathways (*y*-axis) in which they participate. The coloured dots represent the BGC corresponding to each annotated gene.

Metabolic pathway analysis using KEGG [71] indicated that six genes from four novel BGCs are involved in at least seven metabolic pathways, including metabolism, fatty acid metabolism, fatty acid biosynthesis, vitamin B6 metabolism, diverse antibiotic biosynthesis, secondary metabolite biosynthesis and cofactor biosynthesis (Fig. 5).

In the newly discovered aryl-polyene and ladderane BGCs, two genes (*ctg8834_6* and *ctg576_1*) from Abg1_15 m and Abgc2_21 m and two genes (*ctg4577_1* and *ctg7482_2*) from Lbgc1_15 m and Lbgc2_15 m, respectively, encode 3-oxoacyl-[acyl-carrier-protein] synthase II (EC:2.3.1.179). This enzyme is involved in general metabolism, fatty acid metabolism, fatty acid biosynthesis, vitamin B6 metabolism, as well as fatty acid and cofactor biosynthesis [147,148] (Fig. 5, Table S5). In addition, *ctg4577_2* from Lbgc1_15 m encodes an uncharacterized acyl carrier protein (ACP) (Table S5), a key enzyme central to type II polyketide biosynthesis and other secondary metabolite pathways (Fig. 5). ACPs shuttle intermediates between catalytic partners and play pivotal roles in the biosynthesis of complex and pharmacologically relevant molecules. The identification of an ACP gene within a novel BGC highlights the potential of this cluster to produce unique bioactive compounds, underscoring the biotechnological and pharmaceutical importance of this discovery [149,150]. Notably, this enzyme has been reported to be involved in transporting precursors for aurachin A biosynthesis, an antimicrobial prenylated quinolone alkaloid (Fig. S7) [151,155]. ACPs are crucial to fatty acid and biotin synthesis, and studies have demonstrated the interconnection of these pathways, suggesting a high degree of metabolic adaptability and efficiency [156,157]. The identification of this ACP in a novel BGC emphasizes the necessity for ongoing research to elucidate its functional role in secondary metabolite biosynthesis and its potential applications in drug development [150,158].

The identification of novel BGCs across various metabolic pathways highlights protein promiscuity where enzymes can interact with multiple substrates to produce a diverse range of metabolites [159,160]. This enzymatic flexibility allows a single BGC to generate compounds with distinct structures and activities [161]. For example, in *Burkholderia thailandensis*, one BGC produces two variants of hydroxamic acid quinolines [162]. Similarly, a single BGC is responsible for the biosynthesis of ferroverdines and bagremycins in *Streptomyces lunaelactis* [163].

Moreover, phylogenetic analysis of Lbgc1_15 m and Lbgc2_15 m indicates their relation to a clade containing four known antibiotic metabolites (Fig. 4b), suggesting that these ladderane BGCs could potentially synthesize pharmacologically relevant metabolites. Thus, our findings suggest the synthesis of previously unknown compounds, offering significant potential for industrial and pharmaceutical applications. This highlights the value of preserving and exploring extreme environments, such as cenotes, as promising sources of novel bioactive compounds.

### Aryl-polyenes and ladderanes: uses and applications in biotechnology

#### Aryl-polyenes

Aryl-polyenes are particularly notable for their antioxidant properties, which protect bacteria from the harmful effects of reactive oxygen species and contribute to biofilm formation. These bioactive properties open a wide range of potential applications. As antioxidants, aryl-polyenes could be used to extend the shelf life of polymers and food products by inhibiting oxidation and degradation [94]. Their role in biofilm formation is also of great interest. Recent research has shown that manipulating the BGCs responsible for aryl-polyene biosynthesis in uropathogenic bacteria, such as *E. coli,* can inhibit biofilm formation [93]. This ability to interfere with biofilm formation could be exploited in the development of novel therapeutic strategies against resistant biofilm-forming bacteria. Additionally, studies on *Acinetobacter baumannii*, a nosocomial pathogen with high antibiotic resistance, have revealed that pathogenic strains possess aryl-polyene BGCs, whereas non-pathogenic strains do not. This suggests that aryl-polyenes may play a critical role in the virulence [164].

Another promising area of research involves the genetic manipulation of aryl-polyene BGCs in plant symbionts. These metabolites are crucial for bacterial colonization of plant roots, and modifying the corresponding BGCs could enhance aryl-polyene production, thereby improving plant resistance to environmental stresses [92,165].

#### Ladderanes

Ladderanes, due to their unique molecular structure, offer significant potential for materials science applications. The rigidity and well-defined lengths of ladderane chains make them ideal molecular scaffolds for the synthesis of new materials with tailored properties [166]. Ladderanes, due to their unique molecular structure, offer significant potential for materials science applications. The rigidity and well-defined lengths of ladderane chains make them ideal molecular scaffolds for the synthesis of new materials with tailored properties [108,167].

Our study highlights the exceptional microbial diversity of cenotes and the urgent need to protect these unique ecosystems. The identified BGCs, responsible for producing different types of secondary metabolites like aryl-polyenes and ladderanes, underscore the immense biotechnological potential hidden within these microbial communities. Protecting these environments is essential, as preserving their microbial life is crucial not only for local biodiversity but also for broader ecological stability [168,169]. Given the increasing threats to cenotes, safeguarding their microbial diversity is vital to unlocking novel compounds with applications in medicine, agriculture and materials science.

## Conclusions

Our study provides robust evidence of the remarkable microbial diversity in the coastal cenotes of the Yucatán Peninsula. Using metagenomic analysis of sediment samples, we identified novel aryl-polyene and ladderane BGCs linked to the production of novel bioactive compounds. These unique ecosystems harbour a rich microbial community, reinforcing the potential of cenote sediments as promising sources of novel biotechnological products. The findings demonstrate the vast and largely untapped biotechnological potential of cenote microbiomes, with applications in materials science, medicine and agriculture. However, further research is needed to explore other microbial groups and viruses, uncover their roles in secondary metabolite production and better understand the biochemical properties and functions of the identified BGCs. Considering the increasing environmental threats, protecting and conserving these unique ecosystems is critical to ensure the sustainability of future bioprospecting efforts and support the discovery of novel compounds.

## supplementary material

10.1099/mgen.0.001382 Uncited Supplementary Material 1.

10.1099/mgen.0.001382 Uncited Supplementary Material 2.

## Abbreviations

ACP: acyl carrier protein
BGCs: biosynthetic gene clusters
KEGG: Kyoto Encyclopedia of Genes and Genomes
KS: ketosynthase
MAGs: metagenome-assembled genomes
NCBI: National Center for Biotechnology Information
NRP: non-ribosomal peptide
OM: organic matter
PKS-II: polyketide synthase
SRA: sequence read archive
SRB: sulphate-reducing bacteria
SZ: sedimentary zones
TOC: total organic carbon
