# Supplementary material for: Exploring the microbial communities in coastal cenote and their hidden biotechnological potential
Source: Microb Genom. 2025 Apr 3;11(4):001382. doi: 10.1099/mgen.0.001382 (PMC11968836; doi:10.1099/mgen.0.001382)
Supplement: Uncited Supplementary Material 1. [file mgen-11-01382-s001.pdf]

Exploring the microbial communities in coastal cenote and their hidden biotechnological potential

Perla A. Contreras-de la Rosa[1], Susana De la Torre-Zavala[2], Aileen O'Connor-Sánchez[1], Alejandra Prieto-Davó[3*], Elsa B. Góngora-Castillo [4,5*]

[1] Unidad de Biotecnología, Centro de Investigación Científica de Yucatán. Calle 43 No. 130. Col. Chuburná de Hidalgo, 97205. Mérida, Yucatán, México.

[2] Facultad de Ciencias Biológicas. Instituto de Biotecnología, Universidad Autónoma de Nuevo León, 66425. San Nicolás de los Garza, Nuevo León, México.

[3] Unidad de Química-Sisal, Facultad de Química. Universidad Nacional Autónoma de México. 97356. Sisal, Yucatán, México. apdavo@unam.mx

[4] CONAHCYT- Unidad de Biotecnología, Centro de Investigación Científica de Yucatán. Calle 43 No. 130. Col. Chuburná de Hidalgo, 97205. Mérida, Yucatán, México. elsa.gongora@cinvestav.mx

[5] Current Address: CONAHCYT-Departamento de Recursos del Mar. Centro de Investigación y de Estudios Avanzados del Instituto Politécnico Nacional. Km 6. Antigua carretera a Progreso. Cordemex, 97310. Mérida, Yucatán, México.

Supplementary figures material

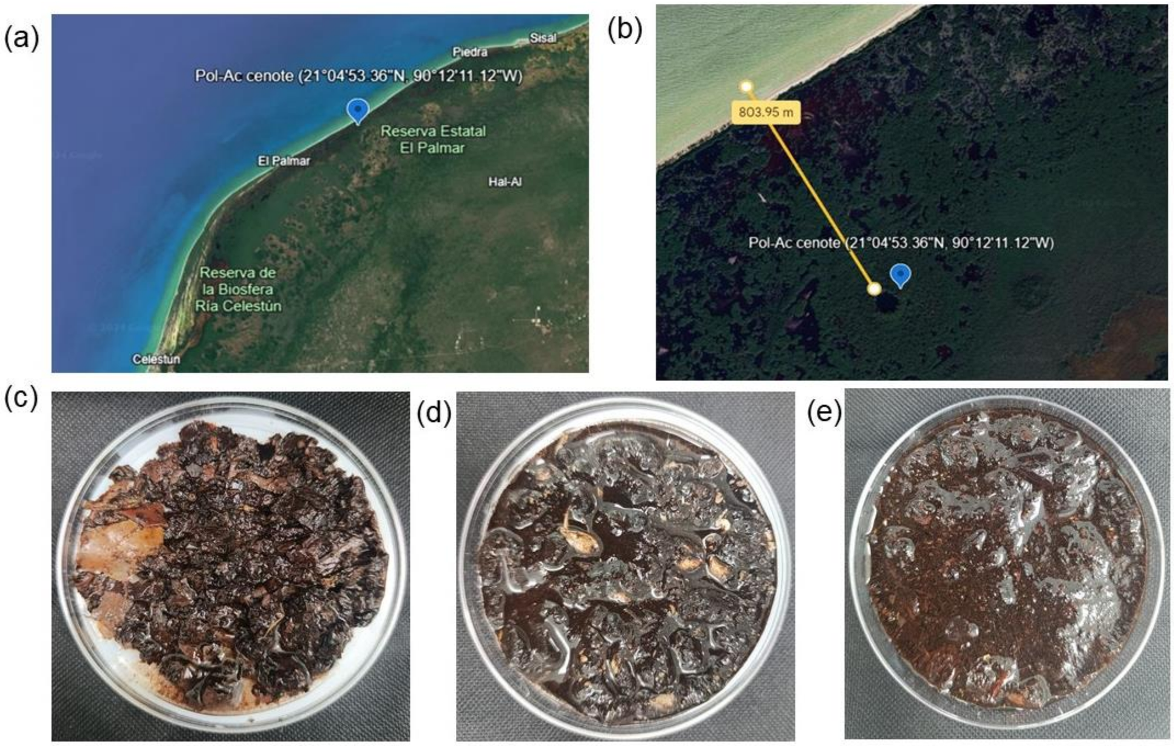

Fig. S1. Sample site and physical characteristics of sediment samples. The upper section shows “Pol-Ac” location maps and the lower section shows sediment images. (a) A map showing geographical location of the Pol-Ac cenote within the ecological reserve “El Palmar.” (b) A map depicting the distance between the Pol-Ac cenote to the coastline. (c–e) Images of sediment samples collected at different depths: (c) sediments from 15 m (SZ15m), (d) sediment from 21 m (SZ21m), and (e) sediment from 54 m (SZ54m). The sediment samples are shown in petri dishes to highlight their physical appearance.

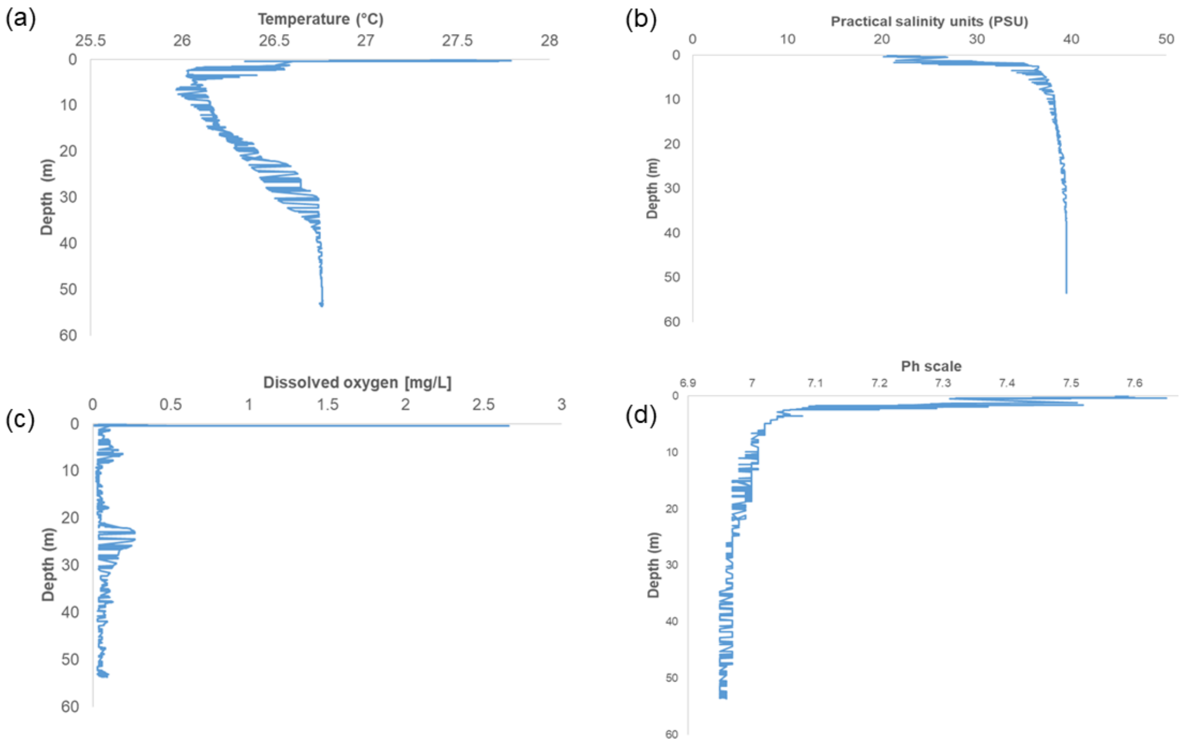

Fig. S2. *In situ* parameters measured per meter of depth, including (a) temperature, (b) salinity, (c) dissolved oxygen, and (d) pH, quantified within the water column (0-54 m) of the Pol-Ac cenote.

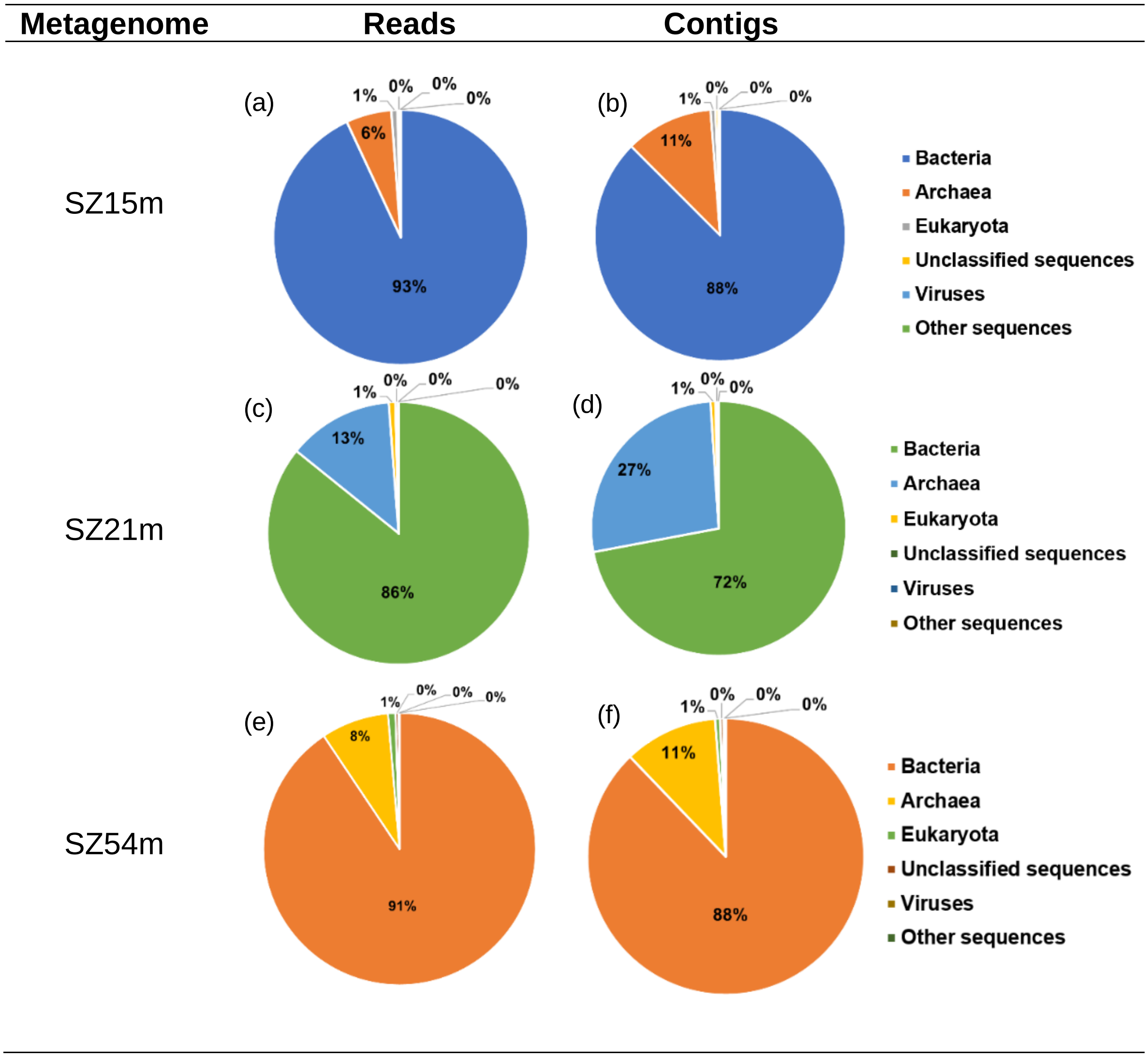

Fig. S3. MG-RAST report showing the relative abundances at the domain level for taxonomically annotated reads and contigs of microbial communities. (a-b) SZ15m metagenome, (c-d) SZ21m metagenome, and (e-f) SZ54m metagenome.

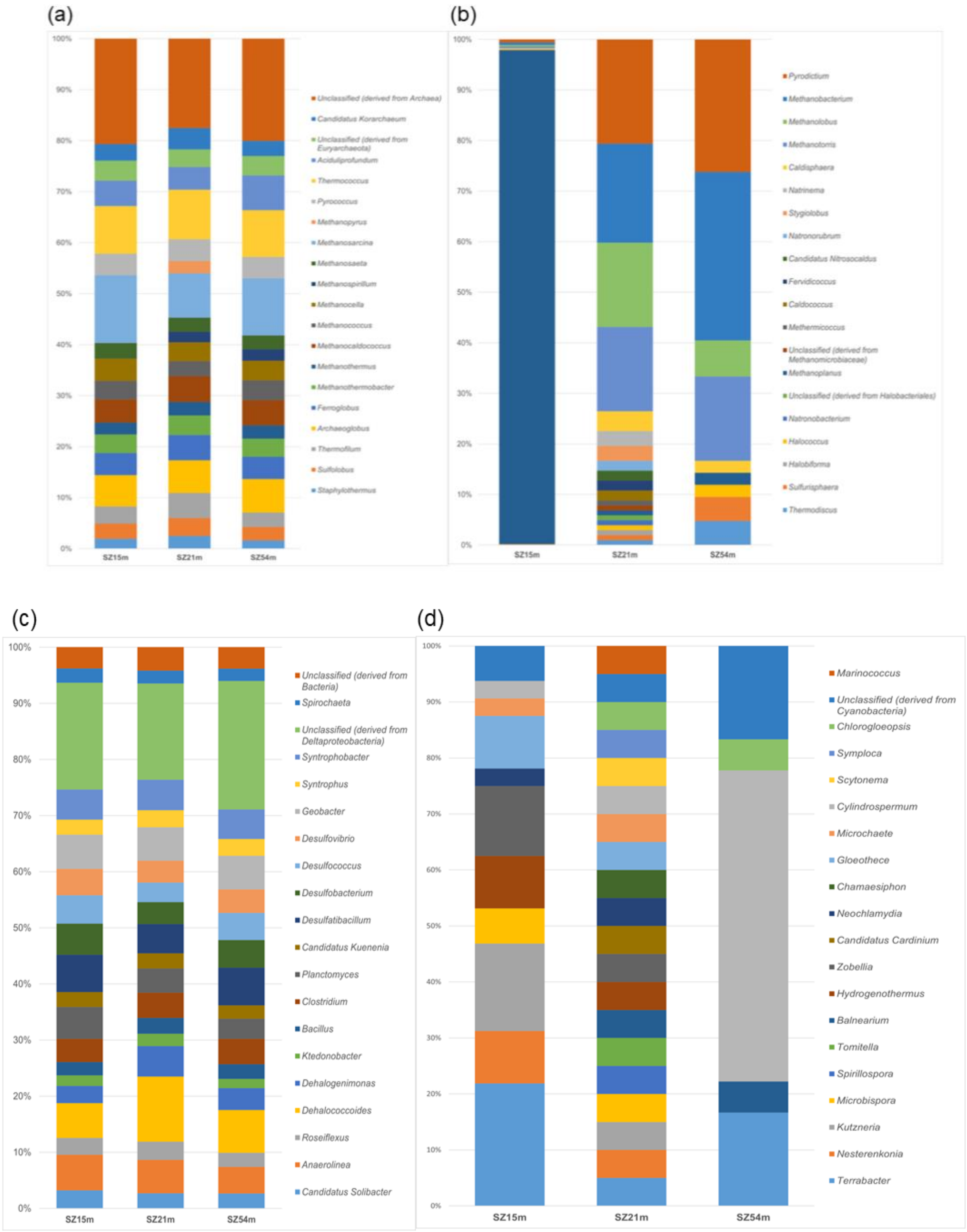

Fig. S4. Relative abundance of the 20 most abundant archaeal (a, b) and bacterial (c,d) genera at different depths. Less abundant genera are considered those with a relative abundance of 1%.

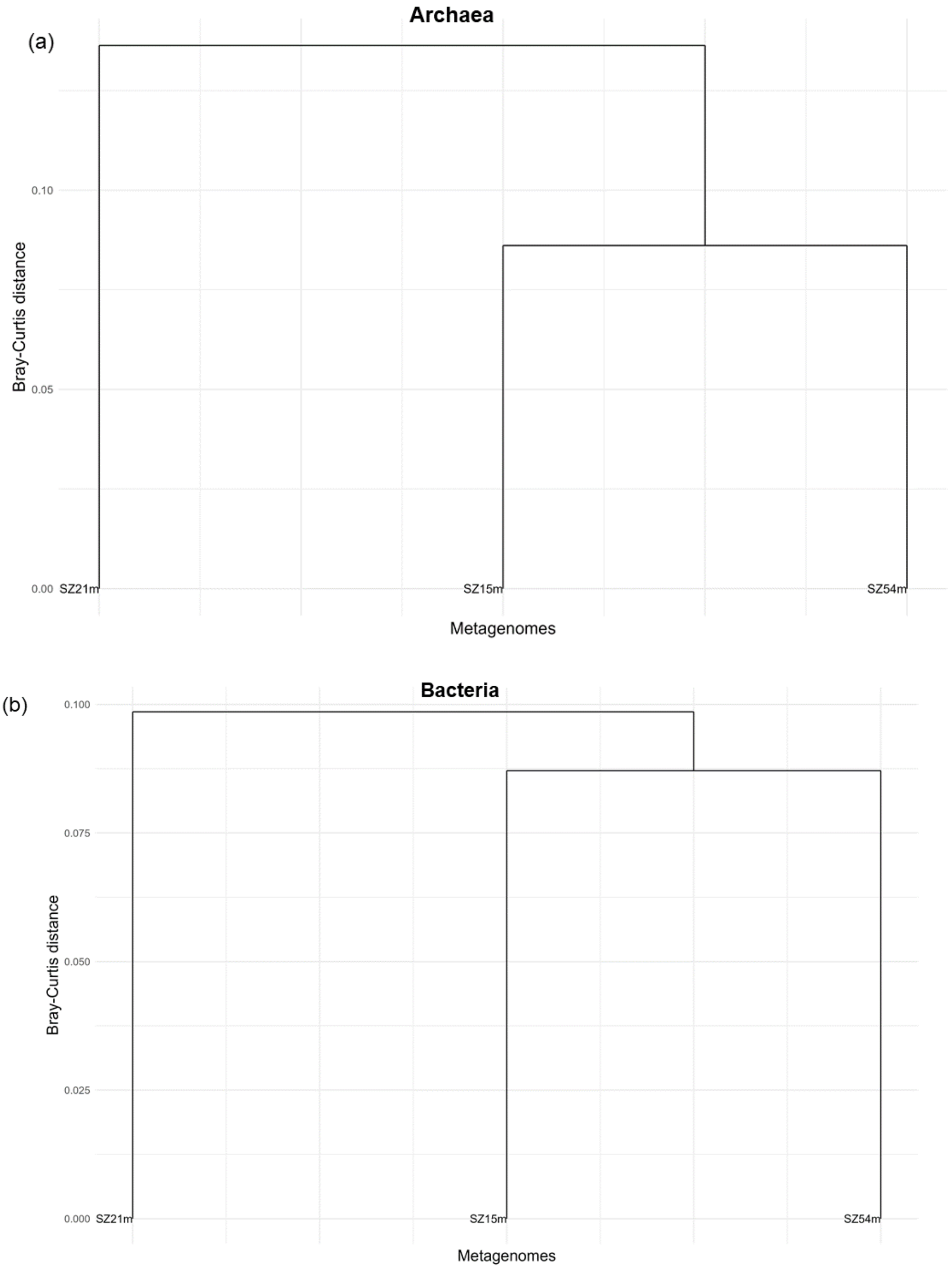

Fig. S5. Bray-Curtis dissimilarity index for bacterial (a) and archaeal (b) genera at different depths.

Metagenome	Bacterial and Archaea contigs
SZ15m	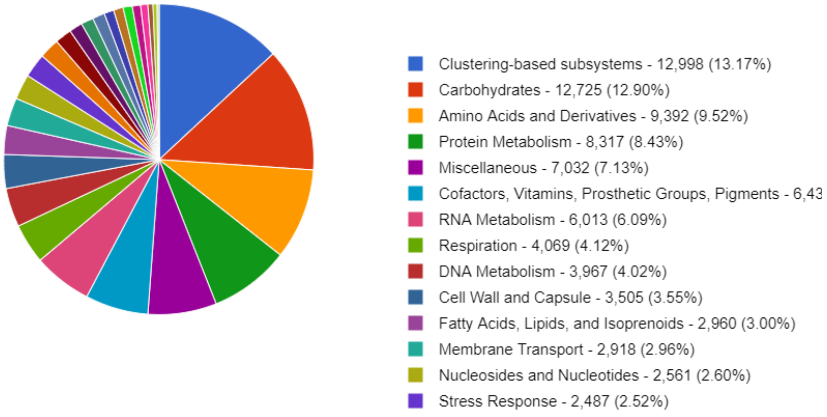
SZ21m	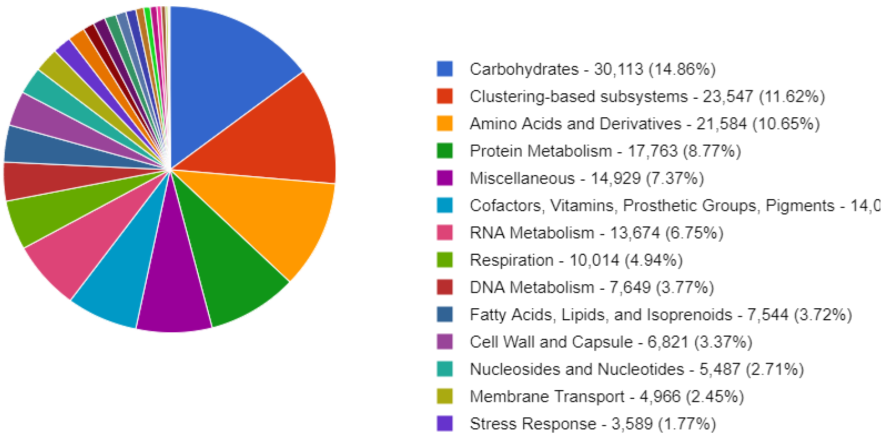
SZ54m	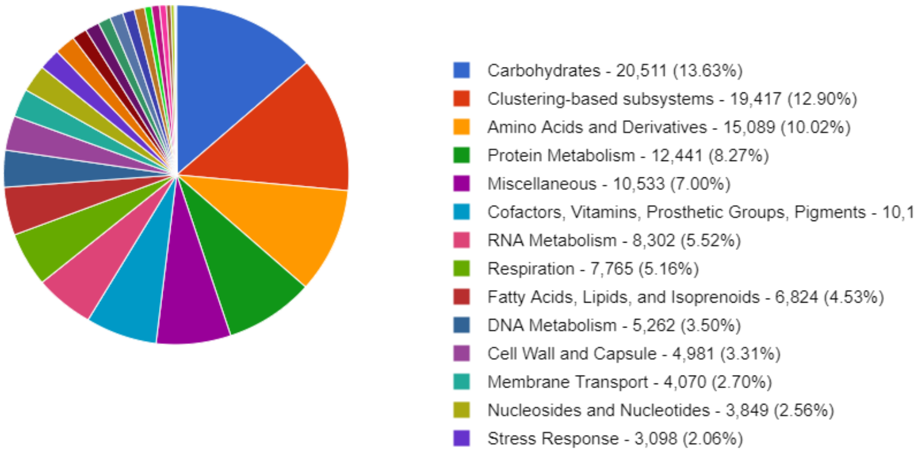

Fig. S6. Different functional hit category using KEGG Orthology (KO) database sub-system annotation.

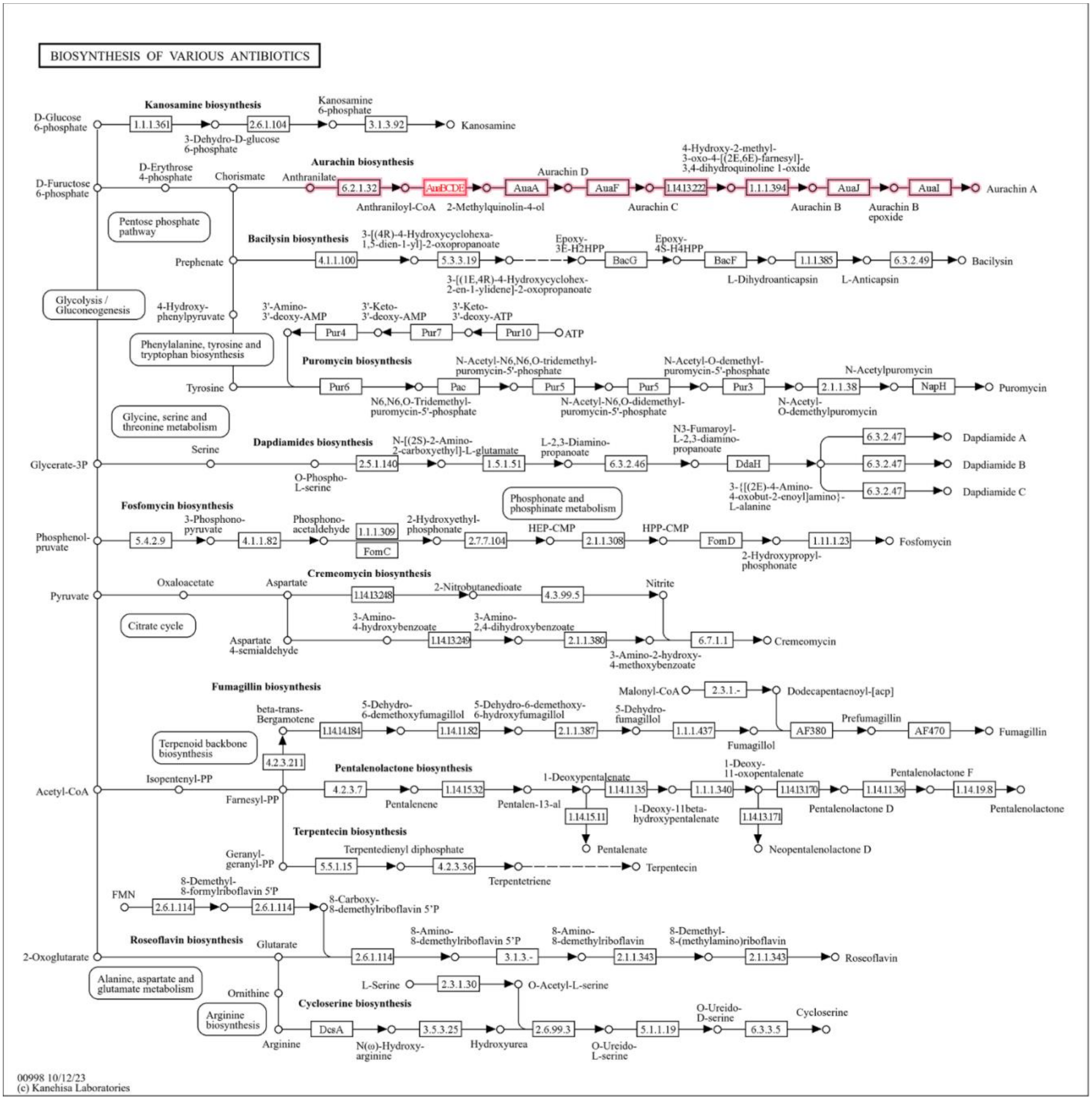

Fig. S7. Map of the biosynthetic pathways for various antibiotics, highlighting the involvement of the ctg4577_2 gene within the BGC Lbgc1_15, specifically in the biosynthetic of Aurachin A. The pathway associated with Aurachin A is highlighted in red.

References

Oksanen, J. et al. Vegan: Community Ecology Package. R Package. 2022. Available from: https://cran.r-project.org/web/packages/vegan/vegan.pdf

Kanehisa M, Furumichi M, Sato Y, Kawashima M, Ishiguro-Watanabe M. KEGG for taxonomy-based analysis of pathways and genomes. Nucleic Acids Res. 2023 Jan 6;51(D1):D587–92.

Kanehisa M, Sato Y, Morishima K. BlastKOALA and GhostKOALA: KEGG Tools for Functional Characterization of Genome and Metagenome Sequences. J Mol Biol. 2016 Feb;428(4):726–31.
